# Lateral Soft Tissue X-ray for Patients with Suspected Fishbone in Oropharynx, A thing in the past

**Published:** 2015-11

**Authors:** Ali Sanei-Moghaddam, Amin Sanei-Moghaddam, Sara Kahrobaei

**Affiliations:** 1 *Department of Otolaryngology, Addenbrookes Hospital, Cambridge, UK.*; 2 *Magee-Women’s Hospital of UPMC, University of Pittsburgh, USA. *; 3 *Burke Mountain Medical Centre, BC, Canada.*

**Keywords:** Lateral soft tissue X-ray, Fishbone, Fibreoptic Nasendoscopy (FNE), Foreign body in the throat.

## Abstract

**Introduction:**

Fishbone is the most common foreign body found in the oropharynx. Conventionally patients with suspected fishbone in the throat would have mirror laryngoscopy followed by lateral soft tissue X-ray to look for the fishbone or observe impacts caused by the fishbone i.e. soft tissue swelling or air in upper esophagus. However, the most common site of fishbone impact is the suprahyoid area, which contains high soft tissue and bony density. This makes X-rays less reliable, especially because not all fish have radio-opaque bones. With the advent of fibreoptic nasendoscopy (FNE) and improved access to CT scan, more reliable tools exist to treat patients with suspected fishbone in the oropharynx;

**Materials and Methods:**

A retrospective study, looking at 698 lateral soft tissue X-rays was performed. This study was conducted in Addenbrookes Hospital, Cambridge (UK) between December 1st, 2004 and February 28th, 2011 using picture archiving and communication systems (PACS). All the radiology reports were reviewed and all the lateral soft tissue X-ray requests for foreign bodies other than fish bones were excluded.

**Results:**

Of the 698 lateral soft tissue X-rays performed between December 1st, 2004 and February 28th, 2011, only 229 (32.8%) were suspected to involve a fishbone in the throat. Amongst those requested for suspected fishbone injury, only 23 (10%) cases were reported by the radiologist as positive for fishbone. Of the 23 patients with a positive finding on X-ray, 13 had negative FNE and were discharged from the hospital, while 5 had fishbone which were visualized using fibreoptic nasendoscope and removed. One patient had an appointment in order to be reviewed in the clinic, but did not show up. The notes for 4 patients were not found; however, there were no records on the hospital intranet suggesting that they had been to the operating room for an ENT procedure related to fishbone. Therefore, it is fair to assume that either there was no fishbone to be found or it was picked up during fibreoptic nasendoscopy and removed under local anesthesia.

**Conclusion:**

Requesting lateral soft tissue X-ray is not beneficial in cases with a suspected fishbone in the oropharynx when fibreoptic nasendoscope is readily available.

## Introduction

Patients with a suspected fishbone in the throat comprise a significant portion of emergency referrals to the ENT team. There have been various studies on diagnosis and treatment of this group of patients and general agreement is that the best diagnostic measure is direct visualization of the fishbone (1). 

Before the introduction of fibreoptic nasendoscope, the only way of direct visualization was to perform rigid pharyngo-laryngoscopy, which had to be performed under general anesthesia. Hence, other diagnostic measures such as mirror laryngoscopy (indirect laryngoscopy) and lateral soft tissue X-ray were used to avoid an unnecessary invasive procedure. The problem is that neither mirror laryngoscopy nor lateral soft tissue X-ray is a reliable diagnostic tool. As for lateral soft tissue X-ray, not all the fish bones are radio-opaque and when it comes to suprahyoid area, where 90% of fish bones are usually lodged, X-ray becomes less accurate due to the presence of high soft tissue and bony density (2). With the advent of fibreoptic nasendoscope, it is now possible to directly visualize the pharynx and larynx and in some cases to remove the fishbone under local anesthesia. There are already studies on how sensitive and specific lateral soft tissue x-ray is when it comes to fishbone (2,3); however, the question to be answered is whether there is still any role for lateral soft tissue X-ray when fibreoptic nasendoscope is available.

## Materials and Methods

Based on the above argument, a retrospective study looking at all the lateral soft tissue X-rays performed on patients with a suspected fishbone in the throat was conducted in our centre. All the radiology reports were checked and the notes were reviewed for the cases which were reported by a radiologist as suspected for fishbone.

## Results

There were 698 lateral soft tissue X-rays requested between December 1st, 2004 and February 28th, 2011 of which 229 (32.8%) were for a suspected fishbone in the throat. Only 23 (10%) cases were reported by a radiologist as positive for fishbone.

Of the 23 patients with a positive finding on X-ray, 13 had a negative FNE and were discharged from the hospital, while 5 had fishbone which were visualized by FNE and removed. One patient had an appointment to be reviewed in the clinic, but never actually showed up (Table.1).

**Table 1:** patients who had possible fishbone in oropharynx based on the radiology report

**Number of patients**	**Treatment**	**Comment**
13	FNE did not show any fishbone and patient was discharged from the hospital	
1	Left tongue base removed under GA due to the gag reflex. It was picked up on FNE rather than an x-ray.	ENT on call could not pick up the fishbone on X-ray. X-ray was reported 3 days later.
4	Fishbone visualized on FNE and removed under local, no need for X-ray.	x1 in valleculae, x1 tongue base, x1 lower pole of right tonsil.
1	Booked by Emergency Medicine team to attend ENT Emergency clinic, the patient did not attend.	
4	No notes found, neither a discharge letter on the hospital intranet confirming operation nor am outpatient clinic letter was found	

The notes for 4 patients were not found; however, there were no records on the hospital intranet suggesting that they had been to the operating room for an ENT procedure related to fishbone. Therefore, it is fair to assume that either there was no fishbone to be found or it was picked up during fibreoptic nasendoscopy and removed under local anesthesia.

## Discussion

Patients with a suspected foreign body in the throat are amongst the most common emergency referrals to the ENT team and fishbone is the most common foreign body found in the oropharynx (4).The most common sites of impact of a fishbone in the oropharynx are the vallecula, tongue base, and tonsils (5).

Almost all the patients show a similar history of a sharp pain sensation while eating fish. Conventionally, all patients undergo ENT examination including mirror laryngoscopy followed by lateral soft tissue X-ray of the neck. Mirror laryngoscopy is heavily dependent on the examiner’s experience and the patient’s compliance and is not always straight forward. This in turn affects the reliability of the examination; hence there is more tendency to rely on lateral soft tissue x-ray findings. Before the advent of fibreoptic nasendoscope, all patients with a suspected foreign body in the throat, based on ENT examination and X-ray findings, would have a rigid pharyngoscopy under general anesthesia, which not only can cause some complications but also most importantly does not always lead to finding the foreign body (1). The problem with this approach is that lateral soft tissue X-ray is neither a sensitive nor specific test for detecting fishbone in the throat (2). That is due to the fact that: 

Not all fish species have radio-opaque bones and the majority of fishbone impact is in the suprahyoid area (6), which contains maximum soft tissue and bony density. This makes lateral soft tissue X-ray less accurate (2).

There are several case reports discussing the complications of missed fishbone in the throat ranging from retropharyngeal abscess, esophageal perforation, and mediastinitis to death (1,7,8). This has made the otolaryngologists to have low threshold in taking the patients to theatre for direct pharyngoscopy.

With the advent of Fibreoptic Nasendos- copy, all patients with a suspected fishbone can be examined on the ward and only the patients who have a confirmed fishbone in the throat would go for direct pharyngo- scopy under general anesthesia. Considering the fact that the majority of fishbone affect the area above the hyoid bone, there is even a chance to remove the fishbone under local anesthesia, providing that the patient is compliant.

There is an argument that the reason behind requesting lateral soft tissue X-ray is not only to look for the actual fishbone, but also to look for other signs of fishbone impact i.e. air in the soft tissue, soft tissue swelling or gas in upper esophagus. However, it takes between 3-12 hours for these signs to appear on X-ray and patients usually attend the Accident and Emergency Department shortly after the fishbone becomes lodged in their oropharynx (1).

A comparison between lateral soft tissue X-ray of the neck and CT scan showed that CT scan is 100% sensitive and specific in detecting fishbone or signs of the impact of the fishbone in the throat and is overall is more reliable than lateral soft tissue X-ray when it comes to fishbone (3).

## Conclusion

It is therefore suggested that all patients with a suspected fishbone in the throat should undergo full ENT examination including fibreoptic nasendoscopy. If the examination is unremarkable, then the patients should be reviewed in the ENT clinic if they did not notice improvement after 48 hours at which point, the patients should have a CT scan to rule out any suspected fishbone. 
